# Editorial: Microbial interactions across species: shaping pathogenesis, symbiosis, and ecosystem dynamics

**DOI:** 10.3389/fmicb.2026.1810035

**Published:** 2026-03-03

**Authors:** Jong Hyun Ham, Bryan Swingle, Gregg S. Pettis

**Affiliations:** 1 Department of Plant Pathology and Crop Physiology, Louisiana State University Agricultural Center, Baton Rouge, LA, United States; 2 Emerging Pests and Pathogens Research Unit, Robert W. Holley Center, United States Department of Agriculture-Agricultural Research Service, Ithaca, NY, United States; 3 Department of Biological Sciences, Louisiana State University, Baton Rouge, LA, United States

**Keywords:** host-microbe interactions, inter-species interactions, microbial interactions, pathogenic interactions, symbiotic interactions

Across virtually all environments on Earth, diverse microbial populations engage in continuous interactions shaped by mutualism, parasitism, competition, and commensalism. These relationships are driven by molecular signals, metabolite exchange, and horizontal gene transfer, processes which shape microbiome structure and influence host health and ecosystem function. Therefore, understanding microbial interactions in natural environments offers critical insights into the technical advancement in medicine, agriculture, and environment sustainability. This Research Topic provides a forum for advanced information about interspecific microbial interactions across various host environments, featuring seven original research articles and two review articles.

Sundray et al. examined gut microbiome composition and function in male and female catla, a freshwater fish (*Labeo catla*), during active gametogenesis using 16S rRNA V3-V4 sequencing data. In this research, Proteobacteria, Fusobacteria, Bacteroidetes, and Firmicutes dominated the foregut, with *Cetobacterium* and *Shewanella* forming the core genera in both sexes. Nevertheless, alpha- and beta-diversity analysis showed clear sex-specific differences, indicating distinct male and female microbiomes. *Shewanella* and *Serratia* were positively associated with female estradiol levels, suggesting microbial links to reproductive readiness. Despite low abundance, *Clostridium perfringens* and *Pseudomonas stutzeri* were identified as keystone taxa shaping community structure.

Ha et al. demonstrated that the slug-killing activity of *Phasmarhabditis hermaphrodita*, a biological control nematode, is strongly influenced by its bacterial associates. Nematodes reared with their naturally associated strain of *Pseudomonas* sp. killed adult gray field slug *Deroceras reticulatum* significantly faster than those reared with *E. coli* OP50 or with their original mixed bacterial community, though the bacterial community also enhanced killing to a lesser degree. These results indicate that associated bacteria markedly affect nematode pathogenicity. Furthermore, *Pseudomonas*-treated nematodes showed increased abundance of four *Pseudomonas* amplicon sequence variants (ASV) in nematodes from infected slugs, highlighting the potential importance of this genus in nematode virulence.

Hurd et al. examined the interaction between *Helicoverpa zea* (corn earworm) and its commensal bacterium *Enterococcus faecalis*, focusing on the role of *ntpJ* gene, which encodes the KtrB subunit of the KrtAB Na^+^/K^+^ symporter. Based on the hypothesis that osmoregulation and ionic homeostasis support bacterial persistence in the gastrointestinal tract (GIT), the researchers tested whether *ntpJ* contributes to survival in this environment. Their results confirmed that *ntpJ* is essential for the survival and long-term persistence of *E. faecalis* in the corn earworm GIT, supporting its proposed physiological function.

In their review article, Eaker et al. describe the symbiosis between legumes and nitrogen-fixing bacteria, emphasizing how legumes regulate their symbiotic bacteria through antimicrobial peptides. Unlike defensins, nodule-specific cysteine-rich peptides (NCRs) drive rhizobacteria into an irreversible, terminally differentiated bacteroid state, effectively domesticating them for the plant. Production of NCRs are a unique feature of the informal IRLC clade of legumes, with NCR-like peptides also presents in the Dalbergioid clade. This system illustrates how host plants control symbiont physiology through targeted peptide production. The authors also discuss NCRs in terms of their evolution, biochemical properties, gene regulation, and potential application in managing plant and animal pathogens.

Wang et al. investigated the gut microbiome of the soybean stinkbug (*Riptortus pedestrias*), a major pest of soybean, to identify microbial targets for pest management. Shotgun metagenomics of 2^nd^ – 5^th^ instar nymphs and adult females revealed dominance of *Enterococcus* and *Caballerronia*, along with distinct stage-specific microbial communities. Notably, antibiotic elimination of *Burkhoderia* bacteria disrupted normal molting and shortened nymphal lifespan. However, the microbiome also contained numerous antibiotic- resistance genes and resistant bacteria, suggesting that while microbiome disruption may aid pest control, indiscriminate antibiotic use could rapidly select for resistant microbes.

Vidayanti et al. examined the genetic basis of differing cytotoxicity levels displayed by two *Legionella pneumophila* strains toward *Tetrahymena thermophila*, a protozoan host. *L. pneumophila* causes Legionnaires' disease and can survive within this bacterivorous ciliate. Using RNA-seq and targeted gene disruptions, the authors identified genes responsible for the heightened toxicity of strain Ofk308, isolated from a Japanese foot spa, compared with the non-cytotoxic Philadelphia-1. Their analyses highlighted *fosA1* (VOC superfamily enzyme) and *msrB/A* as key determinants of cytotoxicity and intracellular growth, advancing ecological and public health understanding of *Legionella* behavior in man-made water systems.

He et al. reviewed recent advances in linking *Fusobacterium nucleatum* (Fn) to colorectal cancer (CRC), emphasizing its emerging role as both a biomarker and a potential therapeutic target. Integrating mechanistic, multi-omics, and clinical findings, they highlight fecal Fn detection (primarily by qPCR) as a promising non-invasive screening strategy. Fecal Fn levels correlate with tumor stage, lymph-node metastasis, and reduced 5-year survival. Advances in Fn genetics (CRISPRi, transposon mutagenesis) and multi-omics reveal subspecies-specific pathogenicity and therapeutic avenues, such as antibiotics, phage, and metabolic inhibitors. The authors also note gaps in standardized detection, thresholds, and clinical validation.

Ticer et al. examined the autofluorescence phenomenon of *Clostridioides difficile*, a gastrointestinal pathogen that causes pseudomembranous colitis, with a focus on microbial metabolites influencing this phenomenon. They found that co-culturing specific gut microbiota species with *C. difficile* enhanced its autofluorescence. Notably, co-cultivation with the opportunistic gut bacterium *Klebsiella pneumoniae*, combined with flow cytometry, enabled efficient sorting of fluorescent *C. difficile* cells from non-fluorescent *K. pneumoniae*. This study revealed interspecies interactions that elevate *C. difficile* autofluorescence and highlights its potential as a tool for visualizing the bacterium's presence and distribution in experimental systems.

Shigeta et al. explored how the methylotrophic yeast *Candida boidinii* influences the fitness of the plant-growth-promoting yeast *Papiliotrema laurentii* on *Arabidopsis thaliana* leaves through its methanol assimilation and pectin methyl esterase activities. They found that *C. boidinii* enhanced *P. laurentii* colonization on leaf surfaces and increased its growth in pectin media, correlating with the yeast's pectin-degrading methylesterase activity. The authors propose that *C. boidinii* degrades pectin to generate methanol for its own metabolism while producing demethylesterified pectin, which is then utilized by *P. laurentii* to facilitate its phyllosphere colonization.

In conclusion, the papers presented in this Research Topic offer valuable insights into cross-species microbial interactions, highlighting the biological forces that drive inter-kingdom relationships and shape microbiomes across diverse environments. Collectively, this body of work advances our understanding of microbiological processes that influence ecosystem function and provides knowledge useful for agriculture, environmental management, and human health.

